# Experiences of people affected by cancer during the outbreak of the COVID-19 pandemic: an exploratory qualitative analysis of public online forums

**DOI:** 10.1007/s00520-021-06041-y

**Published:** 2021-02-11

**Authors:** Sara Colomer-Lahiguera, Karin Ribi, Hayley J. Dunnack, Mary E. Cooley, Marilyn J. Hammer, Christine Miaskowski, Manuela Eicher

**Affiliations:** 1 Institute of Higher Education and Research in Healthcare (IUFRS), Faculty of Biology and Medicine, University of Lausanne, Lausanne, Switzerland; 2 Department of Oncology, Lausanne University Hospital (CHUV), Lausanne, Switzerland; 3 International Breast Cancer Study Group (IBCSG), Coordinating Center, Bern, Switzerland; 4 School of Nursing, University of Connecticut, Storrs, CT USA; 5 Dana-Farber Cancer Institute, Boston, MA USA; 6 Department of Physiological Nursing, School of Nursing, University of California San Francisco, San Francisco, CA USA

**Keywords:** Oncology, Cancer care, COVID-19, Experience, Qualitative research

## Abstract

**Purpose:**

Studies focusing on patients with and survivors of cancer during the COVID-19 pandemic highlight unique psychological and behavioral challenges. These findings were obtained in surveys using self-report questionnaires with pre-specified response options that may not capture the broad range of experiences of individuals affected by cancer, including people with cancer and informal caregivers, in this unprecedented situation. Online forums produce a large amount of valuable first-hand user-generated content that can be used to better understand their day-to-day lives. This study, based on the analysis of narratives in cancer online forums, aims to describe and categorize the experiences of people affected by cancer during the outbreak of the COVID-19 pandemic.

**Method:**

An inductive, descriptive, thematic approach was applied to publicly available cancer forums from Germany, the USA, the UK, and Ireland posted between mid-March and mid-April 2020.

**Results:**

An analysis of the content of 230 main posts revealed three major themes: (1) concerns related to the impact of COVID-19 on cancer care, the risks and fears of getting infected, logistic issues, and economic impact; (2) adaptation challenges faced at the individual and societal level; and (3) the need for advice including information about COVID-19 and the (self-)management of cancer symptoms and treatment.

**Conclusion:**

Our qualitative description of the experiences of people affected by cancer during the COVID-19 pandemic outbreak can help to improve communication, education, and the development of supportive care strategies. Furthermore, the themes and subthemes identified could potentially inform item development for future self-report questionnaires.

**Supplementary Information:**

The online version contains supplementary material available at 10.1007/s00520-021-06041-y.

## Introduction

With the growth of internet access, online peer support groups or forums have gained popularity and have contributed to the empowerment of people with cancer and informal caregivers (ICs) [1, 2]. Evidence suggests that the use of unsolicited data from the internet is a rich, efficient, and inexpensive source to investigate health-related experiences [3–5]. Online forums have the advantage of not being limited by time, space, or material resources [6] and allow participants to express their feelings and frustrations and address sensitive topics, in contrast to narratives requested by the researcher [5, 6].

The COVID-19 pandemic challenged the care for people affected by cancer. At the outbreak of the pandemic, international and national oncology societies developed recommendations aiming to re-organize cancer centers and reduce hospital visits, admissions, and therapy-related complications without compromising patient outcomes [7]. Anxiety, fear, and psychological distress among patients with cancer were expected to be high [8–11].

The first studies of patients with cancer highlighted the unique psychological and behavioral challenges, as well as resilience of patients with and survivors of cancer during the COVID-19 pandemic [12–16]. Several reports found that more than a third of patients with cancer reported high levels of stress and an extremely high symptom burden during the pandemic [17–19]. These findings were obtained using self-report questionnaires. While this approach can be used to describe patients’ experiences, they are limited by asking predefined questions with pre-specified response options. These questionnaires may not capture the broad range of experiences of individuals affected by cancer. Furthermore, these surveys rarely included ICs and none included people with a suspected diagnosis of cancer.

Online forums produce a large amount of valuable first-hand user-generated content from patients, ICs, and the larger public that can be extrapolated to better understand and address their needs and concerns [5].Therefore, based on the analysis of narratives in cancer online forums, this study aims to describe and categorize the experiences of people affected by cancer during the outbreak of the COVID-19 pandemic.

## Methods

This study was based on a qualitative descriptive approach with no conceptual framework predefining the analysis. We searched for threads related to COVID-19 (keywords #COVID; #CORONAVIRUS; #PANDEMIC) in online forums for people affected by cancer from different countries. We selected threads initiated between mid-March and mid-April 2020. This period corresponds with the outbreak of the pandemic in Europe and the USA [20] (Supplementary Fig. 1) and implied the major changes in cancer care. The selection of the forum sites was based on their public accessibility and, among them, the most active ones. One German- (i.e., Germany) and three English- (i.e., USA, UK, Ireland) speaking online forums met the selection criteria. The forum site structure included two with a general category or board where users could post any topic related to cancer and two with divided categories for different cancer types (e.g., lung, larynx, leukemia, thyroid, prostate, ovarian/gynecological). Given the public nature of the forums, it is possible that users from other countries accessed the sites and participated in the discussions.

In general, only initial posts (posts initiating a thread) were selected and exported into the MAXQDA software [21, 22]. Initial posts contain the main message that trigger the ensuing discussion (thread). Therefore, they are representative of the questions and/or concerns of the participant. The only exception was one German forum that had one thread on COVID-19 that was continued over a longer period and included different types of messages and questions. In order to ensure anonymity, any information that would identify participants was removed. In addition, quotes were searched on Google and edited when necessary to avoid traceability [23].

Posts were extracted from forums by two researchers (SCL and KR) and coded by three independent researchers (KR, ME, and SCL) using a data-driven inductive approach to identify themes from the collected posts without a predefined framework [24]. Data were analyzed using thematic analysis [25]. Due to the nature and brevity of the posts, as well as the heterogeneity of the forum users (people with/suspecting cancer and ICs, diagnosis, age), a descriptive, not interpretative analysis, was used. In order to capture an accurate representation of participants’ explicit language, a semantic approach for coding was used [26]. Categories and themes were discussed periodically with all of the authors in order to gain consensus on the thematic content. The MAXQDA data analysis software was used for raw data storage and data management including process, analysis, tracking, and saving changes to create the audit trail [27].

## Results

**Fig. 1:**
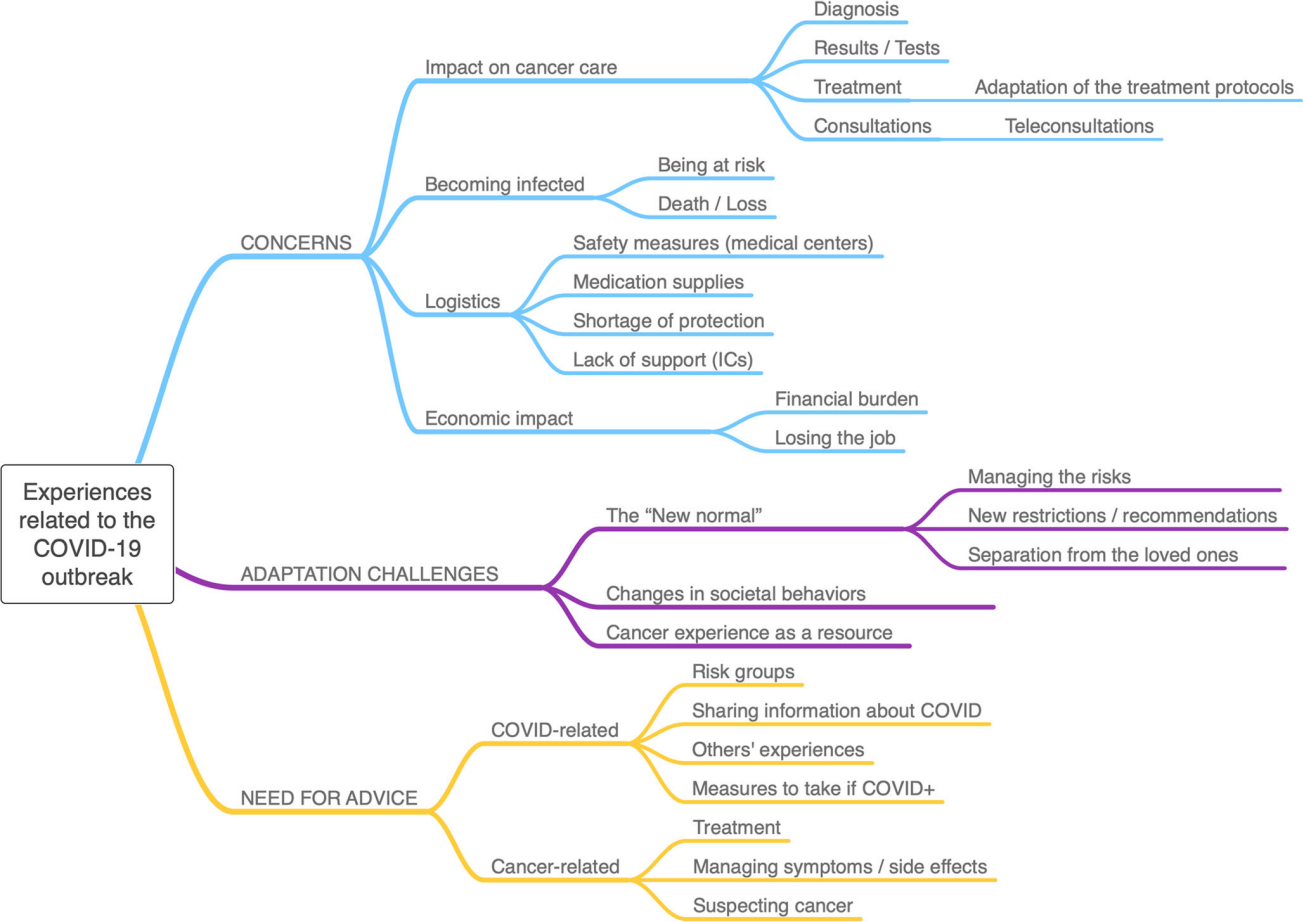
Thematic map of the themes identified representing the experiences of people affected by cancer during the outbreak of the COVID-19 pandemic

We identified 230 main posts, from which 532 segments were coded. Three main themes were identified related to the experiences of people affected by cancer during the outbreak of the COVID-19 pandemic: “concerns,” “adaptation challenges,” and “need for advice”. The attribution of themes was not exclusive, with some posts containing several subthemes. Figure 1 provides a detailed overview of the thematic map, and Supplementary Table 1 describes each theme and corresponding subthemes and codes, with illustrative quotes.

### Theme 1: concerns

#### Impact on cancer care

Users referred to the limited access to healthcare services to get diagnostic tests or to evaluate suspicious symptoms as a main source of their concerns. In particular, they noted that delays may have negative consequences for them: “… because of Covid-19 I am not able to get a scan because the radiologists are helping with the pandemic. I’m really worried. Last night I had a full breakdown and cried because I have no idea what to do.”

Delays and cancelations also applied to treatments and procedures, forcing the rescheduling or adaptation of treatment protocols. Participants experienced these changes as a menace to their health: “I was told I would need 18 [cycles] in total, one every three weeks for a year, but now they are saying we only need nine and will stop in six months. I’m worried this is due to coronavirus and funding; and dropping my treatment by half is a risk to my health.”

One direct effect of the healthcare reorganization was the need to switch to teleconsultations. This transition to telehealth raised concerns about the way examinations could be conducted: “I received a call and was told they were not doing office visits due to Coronavirus, but that they would set up a phone call with my oncologist. I was like “What?!” this is unacceptable as the doctor cannot look for signs of a recurrence from a phone call.” Some participants expressed their worries about not being prepared to participate in a telephone consultation: “… my urgent referral will be a telephone appointment (probably because of coronavirus). I really don’t know what to do. I don’t know how to describe the lump; I personally can’t tell if it is rough edges or smooth.”

#### Becoming infected

Emphasis was placed on the possibility of becoming infected and being at risk due to their conditions or treatment: “Corona has caused me several sleepless hours, the virus is very much on my mind, not only because I belong to a risk group, but also two of my family members have very advanced chronic diseases. That makes my mind wonder.” This concern was perceived by people with cancer as an additional threat that added to the burden associated with their disease and its treatment: “You were already afraid of chemo and now you have to be afraid because you are doubly endangered”.

In addition, concerns about death as a result of getting COVID-19 were present: “I have it in my head that if I became infected I would not survive because of my underlying conditions, age, and everything I hear and read”. Furthermore, the loss of the loved ones, aggravated by the possibility of not seeing them before they died due to the visitor restrictions, was expressed by some ICs: “She may only have little time left with us. The whole COVID thing has destroyed our family communication. We are worried our poor mum might get it and she would be sent to hospital where we may not see her because of the virus.”

#### Concerns related to logistics and supplies

Another major concern was related to logistical issues including the safety measures adopted in the different clinical settings. Going to a medical appointment or to the hospital to receive their treatment was seen as high risk activities: “I am still torn between going there and not going there. I don’t know how Corona-cautious my dentist is. I’ll probably have to flip a coin on Tuesday whether I cancel the appointment or not.”

The possibility of a shortage of personal protective equipment (PPE) (e.g., masks, gloves, disinfectant) was another source of preoccupation: “Yes, it’s scary that we cancer patients can’t protect ourselves when we have to go out into the crowd. Hand sanitizers, disinfectant, wipes, etc. are in short supply.” Likewise, participants expressed problems with availability of some medications and renewal of prescriptions: “At the moment I feel a bit queasy because of the virus, but also because of the shortage of medication.”

Additionally, the pandemic had an impact on the ICs of patients who required special care because of the lack of professional support or the restrictions on hospitals occupancy: “I can’t emphasize enough she is receiving no assistance apart from us until something turns up whatever that may be. How can we ask for care when everyone is on lock down with very limited resources, who is going to help with 24-hour care?”

#### Economic impact

The pandemic had financial consequences for some participants. Expenses related to the stock of supplies for the lockdown and job loss were the main reasons. One IC of a patient that was working as a medical secretary described: “She is afraid she will lose her job. So, while she’s canceling all her own medical appointments out of infection fears, she attends other people’s appointments and sits in the same waiting rooms. Something’s wrong here.”

### Theme 2: adaptation challenges

This theme outlined a series of challenges delineated by participants related to the impact of the COVID-19 pandemic on their daily lives.

#### The “new normal”

These posts described how individuals adapted to the new situation by managing the risks, including the acquisition of PPE; self-isolation or avoiding exposure; and/or the pro-active implementation of risk prevention measures (e.g., working from home, trip cancellations, altering how they shop for groceries): “I do not buy any more bread from the baker, which went through several hands, then they cut it, and money is changed. I do NOT have to have any in this time with my illness.”

Participants used the forums to comment on the new restrictions and recommendations and how they interfered with their plans and routines (e.g., visits, quarantine periods). Moreover, the separation from the loved ones caused by the maintenance of social distancing or the imposition of travel restrictions was a recurrent topic: “I’ve got a little boy and my husband works abroad where he’s stuck due to the Covid-19 outbreak and my parents are in isolation, so I feel so alone and scared.”

#### Changes in societal behaviors

Forum participants mentioned positive and negative changes in peoples’ behaviors and wondered whether interpersonal relationships and solidarity among people will change due to the COVID-19 pandemic. Some shared their observations regarding mistrust among people and egotistic behavior such as compulsive shopping (e.g., toilet paper, yeast) or not adhering to the restrictions: “I am simply speechless, how many people still have not recognized the seriousness of the situation or do not want to recognize it. Here, in the beautiful weather, barbecue parties were celebrated diligently, private soccer tournaments were organized and invitations to birthday parties were issued.”

On the other hand, positive posts were found that described unexpected support received or solidarity experienced. Some of the posts reported that everybody needed to be careful and take measures to not get infected, something that people living with cancer are used to doing. Some posts reminded readers of the things that really matter in life, such as the protection of life, health, cohesion, and charity.

#### Cancer experience as a resource

Some users mentioned that their cancer diagnosis or their experience with the disease and its treatment was a resource to face this new situation. In most of the cases, they expressed their familiarity with physical protective measures and isolation because they had used them previously. The fact of having experienced cancer provided patients with a sense of resilience to face the COVID-19 pandemic: “Strangely enough, that didn’t bother either of us that much. Hey, I’ve got cancer, Corona’s flooding the country... We’ll make it there too. Before I got cancer, I would have been a lot more worried about that.”

### Theme 3: need for advice

The need for advice was a major topic identified in the posts. Users asked others for advice related to COVID-19, cancer, or both.

#### COVID-19 related

One main question focused on individual COVID-19 risk exposure. Participants discussed how to estimate their personal risk or asked others about their risk by sharing information about their cancer, its treatments, or lab values. In addition, they shared thoughts and information about COVID-19 to better understand this unprecedented situation that was experienced as confusing: “I am gradually becoming somewhat confused because I keep reading contradictory statements: Do we as cancer patients have a higher risk or not? During or after chemotherapy of course. But “only” the carcinoma alone? Is there any reliable information?”

#### Cancer related

The other predominant topic was related to the cancer itself and its treatments. Forum participants looked for advice about the adapted treatments; what to do if they did not get treatment due to COVID-19: “Due to Covid-19 my oncologist says oral [drug] is the safest treatment option at the moment with the hope that it will keep me stable until these scary times have passed and more options become available to me. I would love to hear from anyone with a similar diagnosis to find out how their treatment is going”.

A large number of posts consisted of participants sharing signs and symptoms of a suspected cancer that could not be evaluated because of the COVID-19 pandemic: “The fear of a soft tissue sarcoma or any type of cancer is terrifying me, and I would like people to reassure me if it seems overreacting and help me through this time as I cannot see a doctor any time soon due to Covid-19”.

Furthermore, a number of emotions related to the experience of the pandemic appeared throughout the posts. We identified *being scared/fear/panic*, *feeling lost*, *being stressed/anxious*, *being sad/depressed*, *feeling ignored/discarded*, *being upset*, or *feeling alone*, among the most frequent feelings expressed by the forum users.

## Discussion

In this study, we aimed to describe and categorize the experiences of people affected by cancer during the outbreak of the COVID-19 pandemic through a thematic analysis of posts in publicly available online forums. Our results suggest that people affected by cancer who used these forums during the outbreak described how healthcare changes resulted in a variety of concerns and needs related to cancer care and COVID-19. Specifically, delays in diagnosis and treatment, as well as being at risk and becoming infected, were frequent concerns for which forum participants were seeking peer support and advice. Experiences described by participants also included adaptation challenges. First is the need to adapt to a “new normal” characterized by the new restrictions and recommendations and the risk of infection management. Second is the adaptation at the societal level, where many participants perceived the pandemic as a driver of change for both, positive and negative behaviors. Furthermore, some users described their cancer experience as a resource during the early pandemic phase.

The identified themes provide insights into why a relatively high number of people with cancer are reporting clinically meaningful levels of stress and anxiety during the pandemic [12, 13, 17, 18]. In fact, the number of internet searches for *anxiety*, *panic attack*, and *insomnia* significantly increased in the New York area during the COVID-19 lockdown which suggests an overall surge in these symptoms in the general population [28]. More specifically, in a study of oncology patients during COVID-19 [17], a high percentage of patients reported stress levels that were consistent with posttraumatic stress disorder.

Themes and subthemes identified in our study are consistent with some of the themes identified in a previous study in patients with lung cancer during COVID-19 [14]. Specifically, we found similarities in themes related to *changes in oncology practice and access to cancer care* (impact on cancer care), the *perceptions of risk* (becoming infected), or *behavioral and psychosocial responses to COVID-19* (adaptation challenges). In addition, the themes and subthemes “need for advice,” “concerns about logistics” and “financial burden,” or “cancer experience as a resource” provide new insights into the high degree of adversity faced by people affected by cancer, which should be analyzed in future surveys.

Previous research suggests that while both patients and ICs experience loneliness during COVID-19 [15, 29, 30], some patients expressed feelings of societal connectedness because everybody was advised to stay home [15]. This finding is consistent with our subthemes “changes in societal behaviors” and “cancer experience as a resource.” Some participants expressed sentiments regarding isolation and self-protection procedures that could be categorized as being resilient and using positive coping strategies that they learned as part of their cancer experience.

Telehealth has become increasingly predominant during the COVID-19 pandemic. Teleconsultations were encouraged and implemented rapidly in most cancer centers to ensure continuity of care [31]. However, despite all the benefits of teleconsultations (e.g., efficient screening offsite, avoiding travel and exposure [32, 33]), some participants expressed significant concerns about the efficacy and safety of this approach, specifically in terms of the inability to conduct a physical examination. Our findings highlight some improvements that can be made to enhance a telemedicine visit. For instance, people with cancer need education regarding how to perform a virtual consultation, how to use electronic devices, and how to describe signs and symptoms [33].

During a lockdown, the internet and social media are a major source of information [34]. However, because these sources are not always reliable, they may increase fear- and stress-related disorders [34, 35]. Cancer online forums represent a virtual place to share and seek information and emotional support from peers going through or who have had similar experiences [2, 36]. During the COVID-19 pandemic, people affected by cancer described their preoccupations and concerns about restricted access to healthcare services and how these changes would affect their care and outcomes. People with cancer faced the dilemma of going to the cancer center and potentially being exposed to the virus versus postponing or cancelling their treatment to avoid exposure and having their treatment outcome compromised. This finding is consistent with previous studies that noted that treatment delays or interruptions, as well as concerns related to COVID-19, were the dominant factors associated with fears of disease progression, as well as higher levels of anxiety and depression [13, 30, 34, 37]. In addition and related to the limited access to the healthcare system, an important number of threads corresponded to people suspecting symptoms related to cancer, who sought advice or similar experiences from their peers. We believe that the themes identified in this paper can be used by clinicians when they assess patients’ fears and concerns and provide education and self-management support [38].

Limitations of this study include a possible bias toward individuals who seek support and advice from internet sources. However, due to the social distancing limitations imposed by COVID-19, many individuals who do not use online forums regularly may have viewed this approach as an option to obtain information. Furthermore, specific characteristics of the forums’ users (e.g., age, gender, diagnosis, nationality) were not collected in order to maintain privacy. As previously described [21], these two factors constrain the generalizability of our findings. Another limitation is the potential for cultural differences in the use of the forums. While posts from four different countries were evaluated, three of them were English-speaking and represented mainly Western societies. We further acknowledge that the analyzed period of 4 weeks represents a snapshot, corresponding to the outbreak of the COVID-19 pandemic in the four different countries. However, taking into account the evolution of the pandemic, these experiences may change over time.

## Conclusion

Qualitative studies that explore the real-life experiences of patients and ICs during the COVID-19 pandemic are scarce. Public online cancer forums provided a venue for people affected by cancer to share their experiences virtually during the COVID-19 pandemic. The themes identified in this study, “concerns,” “adaptation challenges,” and “need for advice”, provide insights into the impact that the pandemic has had on cancer care and emotional well-being. The results of this study provide new knowledge on the issues that people with cancer and ICs are dealing with during the pandemic. Clinicians can use these topics to improve communication and education of people affected by cancer and as a result, provide optimal supportive cancer care. Furthermore, these results have the potential to inform item development for future self-report questionnaires to be used in surveys during or after this pandemic.

## Supplementary information

ESM 1 (PDF 2713 kb)

ESM 2 (DOCX 22 kb)

## Data Availability

Not applicable
