# Supplementary material for: Experiences of people affected by cancer during the outbreak of the COVID-19 pandemic: an exploratory qualitative analysis of public online forums
Source: Support Care Cancer. 2021 Feb 11;29(9):4979–85. doi: 10.1007/s00520-021-06041-y (PMC7878172; doi:10.1007/s00520-021-06041-y)
Supplement: Supplementary file 1 — (PDF 2713 kb) [file 520_2021_6041_MOESM1_ESM.pdf]

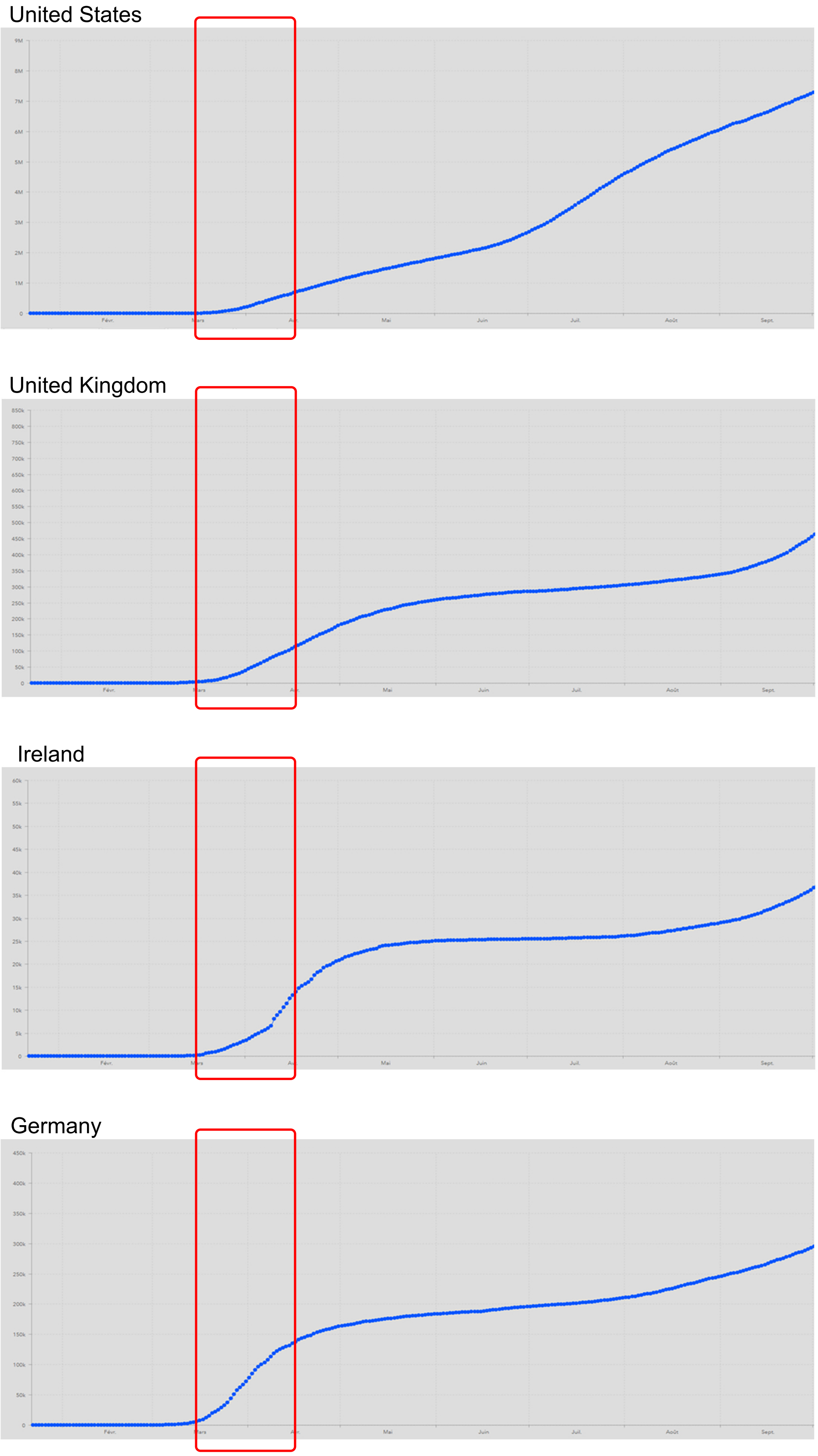

Supplementary figure 1: graphical representation of the cumulative cases per country since 22.01.2020 to 01.10.2020. Red rectangles indicate the timeframe (mid-March to mid-April) used to select main posts on public online cancer patients' forums, corresponding to the outbreak of the COVID-19 pandemic in each country.

Source: COVID-19 Dashboard by the Center for Systems Science and Engineering (CSSE) at Johns Hopkins University
Johns Hopkins Coronavirus Resource Center
https://coronavirus.jhu.edu/map.html (last time accessed: 23.10.2020)

Reference: Dong E, Du H, Gardner L. An interactive web-based dashboard to track COVID-19 in real time. *Lancet Inf Dis*. 20(5):533-534. doi: 10.1016/S1473-3099(20)30120-1"
