# Supplementary material for: Experiences of people affected by cancer during the outbreak of the COVID-19 pandemic: an exploratory qualitative analysis of public online forums
Source: Support Care Cancer. 2021 Feb 11;29(9):4979–85. doi: 10.1007/s00520-021-06041-y (PMC7878172; doi:10.1007/s00520-021-06041-y)
Supplement: Supplementary file 2 — (DOCX 22 kb) [file 520_2021_6041_MOESM2_ESM.docx]

Supplementary Table 1: Themes, subthemes, codes and selected quotes of the experiences of people affected by cancer during the outbreak of the COVID-19 pandemic

| **THEME** | **SUBTHEME** | **CODES** | **SELECTED QUOTES** |
| --- | --- | --- | --- |
| **CONCERNS** | **Impact on cancer care** | Diagnosis | *I have had a swollen lymph node behind my ear for 5 months, it is quite hard and painless. I have been to the doctors about 5 times had blood tests done which came back normal. But because of Covid 19 I am not able to get a scan because the radiologists are helping with the pandemic. I'm really worried. Last night I had a full breakdown and cried because I have no idea what to do.* |
|  |  | Consultations | *How can the oncologists simply say that they are not seeing patients?  I mean, weight loss is a significant sign, and the first thing to do is to check for a local recurrence with an internal exam! It's a bad time to be sick.* |
|  |  |  | *I received a call and was told they were not doing office visits due to Coronavirus, but that they would set up a phone call with my oncologist. I was like "What?!" this is unacceptable as the doctor cannot look for signs of a recurrence from a phone call.* |
|  |  | Teleconsultations | *my first checkup since remission will be in few months and I have the feeling it'll be cancelled due to covid (…) if it's a phone call I worry there won't be that physical examination aspect.* |
|  |  |  | *I got a letter saying that my urgent referral will be a telephone appointment (probably because of coronavirus). I really don't know what to do. I don't know how to describe the lump, I personally can't tell if it is rough edges or smooth.* |
|  |  | Treatment | *Worried that COVID19 will delay my treatment.* |
|  |  |  | *Radiotherapy was planned to start first week in April but had a call saying this is now delayed by two weeks. I was going to start taking an oral treatment after my radiotherapy but wondering if I should start now. Particularly as there is so much uncertainty at the moment. Also thinking there's a possibility it could be delayed further if the situation gets worse.* |
|  |  | Adaptation of the treatment protocols | *Due to delays caused by Covid it could be two or three months before I can have my surgery. I have been put on oral treatment to slow the cancer whilst I am waiting. I have been told I am on the priority list but have already had one date cancelled.* |
|  |  |  | *After the surgery, I received chemotherapy then radiotherapy. Now on my seventh cycle of treatment. I was told I would need 18 in total, one every three weeks for a year, but now they are saying we only need nine and will stop in six months. I'm worried this is due to coronavirus and funding; and dropping my treatment by half is a risk to my health.* |
|  |  | Results / Tests | *Due to the COVID-19 there is a delay on getting the results (…) the waiting process feel like a life time.* |
|  |  |  | *I cannot get [my test] done due to COVID-19 and I feel like slowly losing it. It’s causing me lots and lots of anxiety to the point I start crying randomly.* |
|  | **Becoming infected** | Being at risk | *Corona has caused me several sleepless hours, the virus is very much on my mind, not only because I belong to a risk group, but also two of my family members have very advanced chronic diseases. That makes my mind wonder.* |
|  |  |  | *You were already afraid of chemo and now you have to be afraid because you are doubly endangered.* |
|  |  | Death | *I have it in my head that it I became infected I would not survive because of my underlying conditions, age, and everything I hear and read.  I am contemplating postponing for just 2 weeks and re-assess our local conditions.* |
|  |  | Loss | *I know she may only have little time left with us. The whole covid thing has destroyed our family communication. Because we are worried our poor mum might get it and she would be sent to hospital where we may not see her because of the virus* |
|  | **Logistics** | Safety measures (medical centers) | *I am still torn between going there and not going there. I don't know how Corona-cautious my dentist is. I'll probably have to flip a coin on Tuesday whether I cancel the appointment or not... Oh crap, it would have been easier if they would have been cancelled, then I wouldn't have this decision-problem.* |
|  |  | Medication supplies | *My other big worry is the supply chain as it pertains to prescriptions. I've heard rumblings that it could become an issue as time progresses and I don't see a way to be proactive preparing for that possibility.* |
|  |  |  | *At the moment I feel a bit queasy because of the virus, but also because of the shortage of medication.* |
|  |  | Shortage of protection | *Yes, it's scary that we cancer patients can't protect ourselves when we have to go out into the crowd. Hand sanitizers, disinfectant, wipes, etc. are in short supply.* |
|  |  | Lack of support (ICs) | *When I drive the car I am frightened the police are going to get heavy handed with me for traveling so far. I can’t emphasize enough she is receiving no assistance apart from us until something turns up whatever that may be. How can we ask for care when everyone is on lock down with very limited resources, who is going to help with 24 hour care?* |
|  | **Economic impact** | Financial burden | *We stocked up back in February seeing where things were going. Both my husband and I are retired and this is really hitting our retirement funds!* |
|  |  | Losing the job | *She is afraid she will lose her job. So while she's canceling all her own medical appointments out of infection fears, she attends other people's appointments and sits in the same waiting rooms. Something's wrong here.* |
| **ADAPTATION CHALLENGES** | **The “New normal”** | Managing the risks | *My husband and I have been isolating for eight days in our home, with no outside contact other than grocery deliveries left at our door and then wiped them down before taken inside.* |
|  |  |  | *I do not buy any more bread from the baker, which went through several hands, then they cut it, and money is changed. I do NOT have to have any in this time with my illness.* |
|  |  | New restrictions / recommendations | *I live abroad and because of the Covid-19 restrictions, I cannot fly home. If I did manage to get a flight, would I be able to visit her with a) the quarantine restrictions (14 days self-isolation) that I might be under when I land back and b) the hospital restrictions on no visitors?* |
|  |  | Separation from the loved ones | *I’ve got a little boy and my husband works abroad where he’s stuck due to the Covid19 outbreak and my parents are in isolation so I feel so alone and scared.* |
|  | **Changes in societal behaviors** |  | *I am simply speechless, how many people still have not recognized the seriousness of the situation or do not want to recognize it. Here, in the beautiful weather, barbecue parties were celebrated diligently, private soccer tournaments were organized and invitations to birthday parties were issued.* |
|  |  |  | *But the most important thing is that we all learn again what is really important in life. Namely; health, cohesion, charity, together we are strong.* |
|  | **Cancer experience as a resource** |  | *Strangely enough, that didn't bother either of us that much. Hey, I've got cancer, Corona's flooding the country... We'll make it there too. Before I got cancer, I would have been a lot more worried about that.* |
|  |  |  | *I now read again and again in the media that people are wondering when we can finally live normally again. I think to myself that when I, or rather all of us here, were diagnosed with cancer, there was no return to normality, we already know how it is, not being able to live a carefree life.* |
| **NEED FOR ADVICE** | **COVID-related** | Risk groups | *I am gradually becoming somewhat confused because I keep reading contradictory statements: Do we as cancer patients have a higher risk or not? During or after chemotherapy of course. But "only" the carcinoma alone? Is there any reliable information?* |
|  |  | Sharing information about COVID | *…it was not primarily about the 80% easy cases and not about the 15% severe, not even the 5% most severe cases. Rather, the delay and containment serves to relieve the hospitals. If the number of cases rises sharply, there are no treatment options for the other most seriously ill patients.* |
|  |  | Others' experiences | *I have a question: how do you handle doctor's appointments (check up with oncologist, psychooncologist,etc) during the chemo time? Do you cancel the appointments or postpone them for a few weeks? I would have appointments and would have to go by public transport. These appointments would actually be important, but how to get there?* |
|  |  | Measures to take if COVID+ | *Today I had my fourth chemo in the day clinic. My leukocytes are low. I live together with my husband and two (almost) adult daughters. What do we do if one of them gets corona? Are we all in quarantine then? And what do I do? I wanted to ask this in the clinic, but I forgot to ask it because of my general confusion.* |
|  | **Cancer-related** | Treatment | *Due to Covid-19 my oncologist says oral* [drug] *is the safest treatment option at the moment with the hope that it will keep me stable until these scary times have passed and more options become available to me. I would love to hear from anyone with a similar diagnosis to find out how their treatment is going.* |
|  |  |  | *Is there anybody else having treatment/surgery compromised due to covid 19 and if so how are u coping?!* |
|  |  |  | *My partners’ chemo has been put on hold due to the risk of Covid 19 - is anyone else in this same situation? I am trying to support him but he often feels as if he has being "forgotten" about.* |
|  |  | Managing symptoms / side effects | *I have some significant persistent side effects. I just had my post treatment scan and am waiting for an appointment (Covid delays) to get results. Can someone tell me if the side effects go away? When? Will I ever feel normal again?* |
|  |  | Suspecting cancer | *The fear of a soft tissue sarcoma or any type of cancer is terrifying me and I would like people to reassure me if it seems overreacting and help me through this time as I cannot see a doctor any time soon due to covid-19* |
